# The TAVI Dilemma: Balloon-Expandable or Self-Expanding Transcatheter Heart Valve–Interpreting Current Evidence for Personalized Valve Selection

**DOI:** 10.3390/jcm14165651

**Published:** 2025-08-09

**Authors:** Panayotis K. Vlachakis, Panagiotis Theofilis, Ioannis Kachrimanidis, Stergios Soulaidopoulos, Anastasios Apostolos, Ioannis Skalidis, Paschalis Karakasis, Nikolaos Ktenopoulos, Maria Drakopoulou, Andreas Synetos, Costas Tsioufis, Konstantinos Toutouzas

**Affiliations:** 1 1st Department of Cardiology, “Hippokration” General Hospital, National and Kapodistrian University of Athens, 11527 Athens, Greece; panos.theofilis@hotmail.com (P.T.); iskachrimanidis@gmail.com (I.K.); soulaidopoulos@gmail.com (S.S.); anastasisapostolos@gmail.com (A.A.); nikosktenop@gmail.com (N.K.); mdrakopoulou@hotmail.com (M.D.); synetos@yahoo.com (A.S.); ktsioufis@gmail.com (C.T.); ktoutouz@gmail.com (K.T.); 2 Institut Cardiovasculaire Paris-Sud, Hôpital Jacques Cartier, Ramsay-Santé, 91300 Massy, France; skalidis7@gmail.com; 3 Second Department of Cardiology, Hippokration General Hospital, Aristotle University of Thessaloniki, 54124 Thessaloniki, Greece; pakar15@hotmail.com

**Keywords:** aortic stenosis, TAVI, balloon-expandable valve, self-expandable valve, valve-in-valve, structural heart diseases, interventional cardiology

## Abstract

Transcatheter aortic valve implantation (TAVI) has transformed the treatment of aortic valve stenosis, offering a less invasive alternative to surgical valve replacement, particularly in elderly and high-risk populations. As TAVI expands into younger, lower-risk patients, the choice of transcatheter heart valve has become increasingly important to optimize both immediate and long-term outcomes. Currently, Self-Expandable Valves (SEVs) and Balloon-Expandable Valves (BEVs) are the two most widely used platforms, each characterized by distinct design features, implantation techniques, and hemodynamic profiles. While no definitive evidence supports the overall superiority of one valve type over the other, accumulating clinical data highlight specific advantages and limitations depending on anatomical and procedural contexts. This review aims to present and critically discuss the current evidence, clinical considerations, and evolving concerns surrounding the use of SEVs versus BEVs, with a particular focus on challenging scenarios such as valve-in-valve procedures and long-term valve strategy planning.

## 1. Introduction

Transcatheter aortic valve implantation (TAVI) has revolutionized the treatment landscape for aortic valve stenosis, offering a less invasive alternative to surgical valve aortic replacement (SAVR). As the adoption of TAVI in clinical practice is continuously expanding worldwide, selecting the appropriate transcatheter valve is critical to optimize outcomes and mitigate risk. Guidelines, particularly the European Society of Cardiology (ESC) guidelines, recommend TAVI as a Class I indication for patients with severe, symptomatic aortic stenosis (AS) who are aged ≥75 years (≥65 years in the America Heart Association/American College of Cardiology (AHA/ACC) guidelines), at high or intermediate surgical risk, or unsuitable for SAVR. The Heart Team evaluates clinical, anatomical, and procedural factors to guide informed patient decision-making [1,2].

Currently, Self-Expandable (SEVs) and Balloon-Expandable valves (BEVs) emerge as prominent contenders. While there is no substantial evidence supporting the superiority of the one TAVI device over another, it is important to note that each TAVI device possesses distinctive design and clinical features. The largest trials have been conducted by using BEV and SEV. However, valve selection in clinical practice has largely depended on the implanting team’s familiarity with a particular device and on financial considerations. According to current guidelines and patient populations, these selection criteria may not significantly affect long-term prognosis. Nevertheless, two important trends must be considered: (1) the expansion of TAVI indications to broader patient populations appears inevitable, and (2) the average age of the treated population is decreasing. Moreover, experience with new types of valves is increasing worldwide and technological development provides new valves.

Thus, the selection of the appropriate valve is getting more complex and, according to our knowledge, there is no consensus or specific recommendations. Consequently, selecting the appropriate valve combination presents an additional challenge, facilitating future interventions after the first valve’s degeneration.

This article aims to summarize the key characteristics of two major types of transcatheter heart valves (THV)—the SEV and the BEV—and provide insights into making informed decisions based on the available evidence.

## 2. Self-Expanding Valves: Flexibility and Durability

In 2014, the CoreValve System (Medtronic Inc., Minneapolis, MN, USA) became the transcatheter SEV to receive approval from the U.S Food and Drug Administration (FDA) for the management of patients with severe AS who are at high risk for surgery [3]. Since then, there has been a growing diversity of options available to interventionalists, enabling them to wisely leverage the benefits of each valve according to specific clinical scenarios. There are two self-expanding valve types: supra-annular and intra-annular. Supra-annular SEVs, such as Evolut (Medtronic Inc., Minneapolis, MN, USA) and Acurate Neo (Boston Scientific, Marlborough, MA, USA), typically provide superior hemodynamic outcomes due to larger effective orifice areas (EOAs) and lower transvalvular gradients–especially in patients with small annuli. In contrast, intra-annular SEVs, like Portico (Abbott, Chicago, IL, USA) or Navitor (Abbott Inc., IL, USA), demonstrate hemodynamic profiles more like BEVs but may offer lower rates of conduction disturbances. Hioki et al. demonstrated that Evolut PRO+ (Medtronic Inc., Minneapolis, MN, USA) outperformed BEVs in valve gradients, while BEVs had fewer conduction complications [4]. Chieffo et al. further highlighted that supra-annular SEVs offer better gradients but intra-annular SEVs resemble BEVs in the safety they provide [5].

A major advantage of modern SEVs is the ability to recapture and reposition the valve during the procedure, thereby reducing the risk of malpositioning when compared to the “single-shot” approach of BEVs and older generation SEVs [3]. When dealing with dense annular or subannular calcium, the flexibility and adaptability of SEVs, combined with their consistent radial force, can enhance positioning and anchoring, potentially reducing complications. Caution is required, however, when oversized valves are used, as this has been correlated with higher risk of annular rupture and migration [6]. Moreover, in cases where patients are intolerant to rapid pacing due to factors like low ejection fraction, SEVs offer a more gradual deployment, minimizing hemodynamic instability during the implantation [7].

Patients with small aortic annuli present a significant challenge during TAVI due to the increased risk of elevated transvalvular gradients and prosthesis–patient mismatch (PPM), both of which can adversely affect long-term outcomes [8]. PPM is defined as a condition where the EOA of the implanted valve is too small relative to the patient’s body surface area. Severe PPM (indexed EOA ≤ 0.65 cm^2^/m^2^) has been associated with increased mortality and accelerated structural valve deterioration (SVD). However, the exact threshold for clinical relevance remains debated, particularly in the context of transcatheter valves, where flow dynamics may differ from surgical counterparts [8,9]. SEVs may offer improved hemodynamic performance and a lower incidence of PPM in patients with small aortic annuli, as their valve leaflets are positioned above the native annulus (supra-annular design) [10,11,12]. This design feature contributes to reduced transvalvular gradients and better flow characteristics [13]. The recently published Small Annuli Randomized to Evolut or SAPIEN (SMART) trial compared the outcomes of TAVI in patients with small annuli using SEVs (Evolut PRO/PRO+/FX-Medtronic Inc., Minneapolis, MN, USA) versus BEVs (SAPIEN S3/S3U- Edwards Lifesciences). While there was no significant difference in the primary composite endpoint of death, stroke, or heart failure hospitalization at one year, SEVs demonstrated a significantly lower incidence of bioprosthetic valve dysfunction. This composite endpoint, defined as a mean gradient ≥20 mmHg, severe PPM, at least moderate aortic regurgitation (AR), thrombosis, endocarditis, or the need for reintervention was observed less frequently in SEVs compared to BEVs (9.4% vs. 41.6%, *p* < 0.001) [14]. Long-term data supported these findings. Okuno et al. confirmed that SEVs maintained favorable hemodynamics over five years in small annuli, and Leone et al. further showed that supra-annular SEVs reduced severe PPM—a known predictor of late mortality [9,12]. However, SEVs are associated with higher risk of conduction abnormalities and pacemaker implantation [12]. Given this trade-off, it is reasonable to select a supra-annular SEV for patients with small annuli to achieve better long-term performance.

Adequate transfemoral arterial access is a key element for a successful TAVI procedure. The size of sheath or delivery system that can be used may guide prosthesis selection [15]. A smaller sheath-to-vessel diameter ratio is linked to fewer vascular complications. Data have indicated that SEVs may be a preferred option in patients with smaller arterial diameters, such as females, or in those with challenging iliofemoral anatomy, due to their generally smaller sheath requirements [10]. However, this advantage is not consistent across all SEV systems. For instance, the Evolut PRO+ system (Medtronic) accommodates 23 mm and 26 mm valve sizes with a 14 Fr equivalent sheath, while the 29 mm valve requires a 16 Fr sheath, and the 34 mm valve requires an 18 Fr equivalent sheath. When an introducer sheath is used, the required outer diameter increases further—typically at least 16 Fr for 23 mm and 26 mm valves, 18 Fr for the 29 mm valve, and up to 22 Fr for the 34 mm valve. In contrast, the Navitor system (Abbott) maintains a consistently low-profile design, utilizing a 14 Fr inner diameter sheath for all valve sizes (23–29 mm). This may offer better option for patients with very small or heavily calcified iliofemoral arteries [16]. These differences underscore the importance of individualized valve selection, as not all SEVs provide the same access-related benefits. Patients at a higher risk of bleeding may benefit from the use of SEVs, as demonstrated in the large-scale, propensity-matched CENTER trial, where the implantation of new-generation SEVs was associated with lower incidence of major or life-threatening bleeding compared to new-generation BEVs (2.1% vs. 4.8%, RR 2.3; 95% CI 1.6–3.3, *p* < 0.001) [17]. In addition to this, modern SE devices, such as the Portico or Navitor valve (Abbott Laboratories), also feature improved low-profile delivery systems for patients with limited peripheral access [18].

TAVI is currently increasingly gaining ground for the treatment of younger patients with longer projected longevity, making long-term durability a paramount concern in TAVI. A meta-analysis conducted by Ueyama et al. revealed that the use of SEV was significantly associated with a larger EOA, a lower mean aortic valve gradient, and a smaller increase in mean aortic valve gradient at 5 years in comparison to BEV. Additionally, the investigators noted that SEV use was correlated with lower rates of SVDs when compared to BEV in patients undergoing TAVI [19]. Paravalvular leak (PVL) may occur due to inadequate sizing, malpositioning, or the absence of a sealing region caused by calcification or irregularities due to native valve compression.

Recently, advancements in SEV technology have also extended to the treatment of native aortic regurgitation (AR), a condition that has been historically challenging for transcatheter approaches due to the lack of annular calcification for anchoring. The JenaValve Trilogy system (JenaValve Inc., Irvine, CA, USA), though not yet FDA-approved, represents a novel SEV-based TAVI platform specifically engineered for patients with native AR. It incorporates a self-expanding nitinol frame with a supra-annular leaflet position, porcine pericardial leaflets, and a distinctive locator mechanism that facilitates anatomical alignment and device stability [20]. The system’s large-cell frame enhances coronary access, while its integrated sealing ring aids in achieving annular conformity and reducing PVL. Data from the ALIGN-AR trial support the safety and efficacy of the JenaValve Trilogy in symptomatic patients with moderate or greater AR who are at elevated surgical risk, demonstrating encouraging procedural and short-term clinical outcomes [21]. Furthermore, the JenaValve has obtained CE mark approval in Europe for use in patients with severe symptomatic AS or AR deemed high-risk for surgical intervention.

## 3. Balloon-Expandable Valve: Precision and Control

Mainly represented by the Edwards’ Sapien, BEVs are intra-annular valves that have undergone substantial technological progress, parallel to SEVs in the previous decade. Although higher residual post-procedural gradients have been observed with this type of valve compared to SEVs, this does not seem to be translated into a clinically meaningful outcome with either older or newer generation BEVs.

Undoubtedly, the lack of retrievability and repositionability remains a relative concern, especially for less experienced operators, who can at least take advantage of the shorter learning curve for TAVI with BEVs compared to SEV devices [22]. Data from the FRANCE-TAVI nationwide registry support that the use of BEVs is associated with a lower risk of in-hospital and 2-year mortality compared to SEVs. This may be associated with a lower risk of paravalvular regurgitation, and in-hospital events, such as stroke, myocardial infarction, and pacemaker implantation rates. In fact, among many factors that have been recognized as predictors of new-onset conduction abnormalities, the use of BEVs seems to be accompanied by a 3- to 4-fold lower risk of post-procedural need for permanent pacemaker implantation, which is linked to extended hospitalization, increased costs, and potentially adverse patients’ outcomes. This is most likely attributed to a lower probability of compressing the membranous septum with consequent damage to the cardiac conduction system with BEVs, which makes them a more reasonable selection for patients at high-risk for post-procedural conduction abnormalities [5,17]. Lower rates of periprocedural strokes have also been observed in TAVI performed with BEVs and have been attributed to the substantially shorter implantation time, compared to the slow and stepwise deployment of SEVs. Indeed, increasing the manipulations of the valve system across the calcified aortic annulus and arch before final implantation increases the potential for cerebral embolization of calcified particles in the native aortic valve [23].

Nevertheless, as far as mortality is concerned, the Comparison of Transcatheter Heart Valves in High Risk Patients With Severe Aortic Stenosis: Medtronic CoreValave vs. Edwards SAPIEN XT (CHOICE) randomized clinical trial showed no difference between the two valve types regarding 30-day cardiovascular mortality, despite significant superiority of BEVs in terms of procedure success [24]. The latter was mainly driven by a lower risk for significant AR, especially in patients with large annuli. Nevertheless, there are now new-generation SEVs with special technological features available that allow improved sealing with superior forward hemodynamics [18].

Indeed, the presence of significant PVL is a concerning complication following TAVI and its occurrence has been linked to increased morbidity and mortality. In order to mitigate PVL, newer-generation transcatheter devices have been developed with design enhancements aiming at improved sealing and lower leak incidence. Notably, the Edwards SAPIEN 3 and Medtronic Evolut Pro valves incorporate features such as external skirts or pericardial wraps to enhance paravalvular sealing. Comparisons between these devices have demonstrated that the SAPIEN 3 valve (Edwards Lifesciences) is associated with a lower incidence of moderate or severe PVL compared to the Evolut Pro valve [3]. This suggests a potential advantage of the SAPIEN 3 (Edwards Lifesciences) in minimizing PVL occurrence.

Notably, in addition to the previous, the RESILIA tissue that has been used in the SAPIEN 3 Ultra (Edwards Lifesciences) and SAPIEN X4 (Edwards Lifesciences) valve platforms, has been designed to improve the durability of the bioprothesis by preventing early valve deterioration and calcification [25]. This feature makes the valve particularly appealing for younger TAVI candidates, who would otherwise require reintervention within 8–10 years with the older bioprosthetic valve devices.

In the presence of an extremely horizontal aorta (high angulation), catheter navigation and coaxial alignment can be more challenging with SEVs and, therefore, the implantation of a BEV is usually preferred.

Owing to their low frame height, which allows sub-coronary implantation, BEVs facilitate coronary access post-TAVI, a feature that makes them more suitable for patients at a higher risk for periprocedural coronary artery obstruction, including those with low-lying coronary ostia or narrow sinus of Valsalva, patients with established coronary artery disease or younger patients with a higher likelihood for future coronary interventions [26]. Choosing the appropriate device is even more challenging when it comes to valve-in-valve (ViV) interventions for failed bioprosthetic valves, where a higher risk for coronary obstruction exists. Theoretically, coronary obstruction occurs due to displacement of native valve leaflets or the previous bioprosthetic valve leaflets towards the coronary ostia, or even the positioning of the second bioprosthetic valve frame or commissural suture in front of the coronary ostia [27]. Recent data suggest an association of the transcatheter valve type with coronary obstruction. In the RE-ACCESS trial, an unsuccessful coronary cannulation following TAVI was documented in 7.7% of 300 patients undergoing TAVI and occurred almost exclusively in those receiving a SEV prothesis. A delayed coronary obstruction may also rarely occur with SEV devices, that tend to further expand after the time of TAVI [28]. On the other hand, when the risk of coronary obstruction is considered high, the use of repositionable SEV devices offers immediate assessment of coronary access, so the valve may be retrieved before it is fully deployed, in the case that a coronary obstruction is observed.

**Figure 1:**
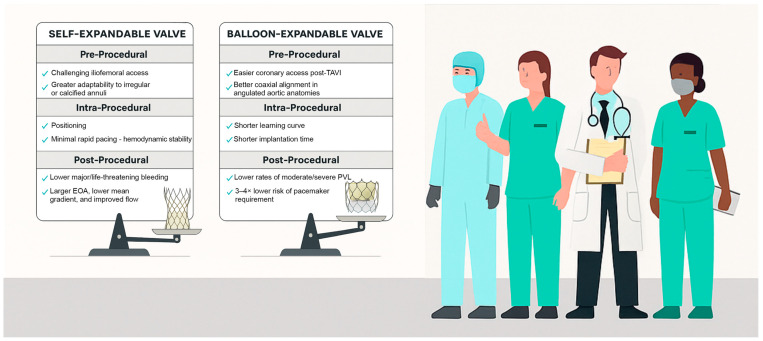
Pre-procedural, intra-procedural and post-procedural advantages of each valve type to achieve optimal TAVI outcomes. Created with BioRender.com.

Figure 1 summarizes the key advantages of each valve type, which the Heart Team should consider adopting a more personalized treatment strategy—moving beyond the “one-size-fits-all” approach to optimize patient outcomes.

Last but not least, while surgical aortic valve replacement remains the first choice for patients with bicuspid aortic valve (BAV) stenosis, the use of BEVs to treat these patients permits improved annular apposition with lower rates of paravalvular regurgitation at one year follow-up in matched cohorts, owing to their higher radial forces, with the cost, however, of a higher risk for procedural annular rupture [29,30]. Recent data from the AD-HOC registry comparing BEVs and SEVs in Sievers type 1 BAV stenosis demonstrated similar technical success and mid-term outcomes, but BEVs were associated with lower rates of new permanent pacemaker implantation and moderate or greater paravalvular regurgitation—at the expense of a higher rate of severe PPM [31]. Similarly, in the BEAT registry evaluating new-generation SAPIEN 3 (Edwards Lifesciences) and Evolut R/PRO devices (Medtronic Inc., Minneapolis, MN, USA), BEVs showed comparable overall safety and success. However, after adjustment, BEVs were associated with significantly less moderate-to-severe PVL at one year, though they also carried a higher risk of annular rupture—again underscoring the importance of device-specific trade-offs [32]. Further support comes from a systematic review and meta-analysis by Ueshima et al., which found that BEVs were linked to fewer second-valve implantations and pacemaker needs, though annular rupture occurred more frequently with BEVs [33]. Importantly, patient-specific BAV morphology significantly influences outcomes, as shown in the International BAV Registry, where patients with calcified raphe and extensive cusp calcification had notably worse two-year mortality [34]. Conversely, favorable results with SEVs have been demonstrated in carefully selected low-risk patients. The Low-Risk Bicuspid study, using Evolut R/PRO (Medtronic Inc., Minneapolis, MN, USA) in patients with limited calcification and low surgical risk, achieved a 95.3% device success rate and only 1.3% early mortality, suggesting SEVs can perform well in optimized anatomies [35].

Together, these findings highlight that BAV represents a highly heterogeneous anatomical substrate. Valve selection in TAVI for BAV patients should therefore be based not only on general device profiles, but also on Sievers classification, raphe calcification, cusp symmetry, and annular dimensions—factors that substantially influence procedural strategy, sizing, and risk of complications.

Table 1 provides a comprehensive comparison of SEVs and BEVs across the procedural continuum, highlighting key factors that inform individualized valve selection and long-term management in TAVI patients.

## 4. Beyond the First Implant: Valve-in-Valve and the Importance of Planning Ahead

As the population undergoing TAVI expands to younger, lower-risk patients, a lifetime valve strategy is becoming increasingly important, as these patients often require multiple interventions over time. Consequently, initial valve selection and implantation techniques are critical to preserving future treatment options. Ensuring optimal valve expansion at the index procedure helps minimize the risk of PPM and valve degeneration, while careful choice of implantation depth is essential to reduce conduction disturbances and pacemaker need, as well as to maintain feasibility for future Valve-in-Valve (ViV) procedures [36]. Within this evolving context, the choice between BEVs and SEVs significantly impacts procedural and long-term outcomes, especially in ViV following failed surgical (TAVI-in-SAVR) or transcatheter valves (TAVI-in-TAVI).

In TAVI-in-SAVR, the anatomic constraints imposed by the surgical valve ring may limit transcatheter valve expansion, particularly in small valve sizes. The supravalvular leaflet position of SEVs offers hemodynamic advantages in this scenario. SEVs tend to achieve larger EOAs and lower residual gradients than BEVs, especially when implanted in small surgical valves. This has been demonstrated in the VIV International Data (VIVID) registry, where post-procedural gradients ≥20 mmHg were more frequent with BEVs (40%) than SEVs (21.3%) in valves with internal diameters <20 mm [37]. These higher gradients have prognostic significance, being independently associated with late mortality [37].

This hemodynamic advantage of SEVs was confirmed in the LYTEN randomized trial, which included patients undergoing ViV with small stented surgical valves (label size ≤ 23 mm). At 30 days, only 21% of SEV cases had a mean gradient > 20 mmHg, compared to 62% of BEV cases, despite a significantly higher use of bioprosthetic valve fracture (BVF) in the BEV group (30% vs. 13%, *p* = 0.04) [38,39]. At 1 year, SEVs maintained superior hemodynamics. However, both platforms demonstrated comparable short-term safety outcomes, though the trial was not powered to detect differences in clinical events.

Coronary obstruction is another major concern in ViV and is influenced by valve type. While the overall incidence is low (~2.3%), mortality can reach 48.6% at 30 days [28]. BEVs may allow safer coronary access due to their shorter frame height and intra-annular leaflet design, particularly when used in anatomies at high risk of obstruction. SEVs, on the other hand, have higher frame profiles and can elevate the leaflets of the failed surgical valve toward the coronary ostia, potentially increasing risk [28,40,41]. The virtual transcatheter-to-coronary (VTC) distance, derived from pre-procedural CT, is a validated predictor of obstruction risk. While simulated distances tend to underestimate actual post-procedure distances in non-postdilated cases, procedural factors like postdilation can reduce final coronary clearance [42].

In high-risk cases, preventive techniques like BASILICA (scallop intentional laceration) or chimney stenting are used, though their long-term safety and comparative effectiveness between valve types remain under investigation [43,44]. Recent innovations, such as the ShortCut device, aim to simplify leaflet modification by mechanically splitting the leaflet without requiring electrosurgical expertise, potentially lowering the technical barrier for operators [45,46].

Another important consideration is the need for permanent pacemaker implantation. In native valve TAVI, SEVs are typically associated with higher pacemaker implantation rates due to deeper implantation and interaction with the conduction system. In ViV procedures, however, the presence of the surgical valve ring provides mechanical protection, resulting in lower overall pacemaker rates. The VIVID registry showed a 6.4% overall permanent pacemaker implantation rate, with Evolut (Medtronic Inc., Minneapolis, MN, USA) SEVs at 3.7% compared to 9% for the earlier-generation CoreValve platform (Medtronic Inc., Minneapolis, MN, USA) (*p* = 0.002), significantly lower than the 17% reported in native TAVI with Evolut valves [47,48].

The long-term outcomes of TAVI-in-SAVR also appear encouraging. The PARTNER 2 ViV Registry reported 50.6% all-cause mortality and 6.6% structural valve deterioration (SVD) at 5 years, aligning with observational data from the VIVID registry [49,50]. Importantly, the PARTNER 3 ViV Registry, which includes lower-risk patients, reported zero mortality at 1 year among 97 patients, though the mean gradient remained relatively high (22.5 ± 8.8 mmHg at 30 days, 19.7 ± 7.6 mmHg at 1 year), with 6.2% requiring reintervention. Half of these reinterventions were associated with valve thrombosis [51]. These elevated gradients reinforce the relevance of valve selection—particularly the advantage of SEV in small annuli.

In the setting of TAVI-in-TAVI, valve selection becomes more complex. Coronary access is often more compromised than in TAVI-in-SAVR due to the “neo-skirt” formed by the displaced prior transcatheter valve leaflets. SEVs, especially those with taller stent frames and commissural misalignment, can make reaccessing coronaries technically challenging. In this setting, a BEV may be preferred for its lower frame and more favorable access profile [40,41]. Conversely, SEVs may be preferred when implanting inside degenerated BEVs, as their supra-annular leaflet position helps optimize hemodynamics, particularly in small BEV frames [38,52].

Despite these anatomical and functional differences, a recent meta-analysis found no significant difference in long-term mortality between BEVs and SEVs in TAVI-in-TAVI [52]. Still, survival benefits may diverge over time. A separate analysis comparing TAVI-in-TAVI with redo surgical aortic valve replacement showed an early advantage for ViV in terms of procedural outcomes—lower 30-day mortality, less stroke, and bleeding—but suggested a crossing of survival curves favoring surgery after two years [53,54]. These findings emphasize the importance of individualized treatment planning, particularly in younger patients with longer life expectancy.

In conclusion, TAVI-in-TAVI is a safe and effective alternative to redo surgical valve replacement, with outcomes influenced by prosthesis type. SEVs offer superior hemodynamics in small surgical valves and may reduce the need for postdilation and BVF. BEVs provide better coronary access and may be advantageous in anatomies with obstruction risk or in TAVI-in-TAVI where reaccess is essential. Careful pre-procedural imaging, an understanding of valve-specific properties, and forward planning for future interventions are essential to optimize outcomes.

## 5. Ongoing Trials

Direct comparisons of events rates between studies evaluating different device types are discouraged due to the significant differences in baseline risk profiles among patient population in various studies. While there is no substantial evidence supporting the superiority of one TAVI device over another, mainly due to the lack of large-scale head-to-head comparisons and the constant development and improvement of existing devices, it is important to note that each TAVI device possesses distinctive design features. Therefore, specific factors related to patients’ anatomies may dictate the use of certain valves. Several ongoing trials have been designed to provide crucial insights into the comparison between BE and SE aortic valves (Table 2). The BASELINE trial (NCT04843072) is evaluating BEV vs SEV systems in the context of failing surgical bioprosthetic valves, aiming to clarify outcomes in this important clinical scenario [55]. The BEST trial (NCT05454150) is the first randomized clinical study designed with a superiority approach, focusing on short- and long-term mortality. It directly compares the mortality benefits of BEVs versus SEVs at both 90 days and 1 year, promising to deliver valuable insights. The CENTER trial (NCT03588247), a large collaborative patient-pooled meta-analysis, will provide extensive data on the impact of BEVs versus SEVs in periprocedural cerebrovascular events. Lastly, the AAD-CHOICE study from China (NCT06009588) will examine the influence of transcatheter heart valve types on intra-procedural device success and post-procedural ascending aorta progression in patients with dilated ascending aortas (≥45 mm) undergoing TAVI, further expanding our understanding of these valves’ effects.

## 6. Future Directions

As the use of TAVI continues to expand into younger and lower-risk populations, future directions in valve technology and procedural strategy are becoming increasingly centered around personalization, durability, and integration with advanced computational tools. The decision between BEVs and SEVs remains a cornerstone of procedural planning, and forthcoming innovations aim to enhance this choice with greater precision and predictability.

One of the most promising developments is the application of digital twin technology, which creates a patient-specific virtual model of the aortic root and valve apparatus. These digital replicas, derived from high-resolution imaging and patient-specific data, allow for simulated valve deployment and hemodynamic assessment. By modeling the unique anatomical interactions of a BEV or SEV with the aortic annulus and surrounding structures, clinicians can predict complications such as paravalvular leak, annular rupture, or conduction disturbances. Such simulations could become vital tools in selecting not just the valve type, but also the optimal size, positioning, and implantation depth.

Artificial intelligence (AI) is expected to further augment this decision-making process. Machine learning algorithms trained on large-scale clinical and procedural datasets may offer real-time, valve-specific recommendations by analyzing anatomical features (e.g., annular dimensions, calcium burden), procedural risk factors, and patient comorbidities. For instance, an AI platform could identify patients at higher risk of post-TAVI pacemaker implantation and recommend a BEV over a SEV, or vice versa in cases where supra-annular valve design might improve hemodynamics in small annuli.

Advances in valve design and materials also continue to shape the future of BEVs and SEVs. BEVs are increasingly incorporating novel bioprosthetic tissues, such as anti-calcification-treated RESILIA, aimed at improving long-term durability [56]. SEVs, on the other hand, are being optimized with repositionable frames, larger cell designs for improved coronary access, and enhanced sealing skirts to reduce paravalvular leak—one of the historical disadvantages of earlier SEV platforms. These structural improvements are particularly relevant for ViV procedures and in anatomically challenging cases such as bicuspid aortic valves or horizontal aortas.

The integration of sensor technology into next-generation valves represents another frontier. Future BEVs and SEVs may include embedded sensors capable of transmitting data on valve performance, pressure gradients, or leaflet mobility. This would enable remote monitoring and early identification of structural valve deterioration or thrombosis—supporting timely clinical intervention.

Finally, the incorporation of robotic-assisted implantation and augmented reality platforms may streamline complex procedures, reduce operator variability and enhance the precision of valve deployment. These tools could prove especially valuable when implanting repositionable SEVs in difficult anatomies or navigating BEVs in cases where coronary access is critical.

Together, these innovations will not only enhance the safety and efficacy of both BEVs and SEVs but will also mark a shift toward a more personalized, data-driven approach to TAVI—aligning procedural strategy with each patient’s unique anatomical and clinical profile.

## 7. Conclusions

In the rapidly evolving landscape of THV technology, SEVs and BEVs stand as prominent contenders. As clinicians, we must strive to select the valve that best aligns with individual patient anatomy, clinical characteristics, and expected outcomes. Collaborative discussions involving multidisciplinary teams, including interventional cardiologists, imaging specialists, and cardiac surgeons, are invaluable for a comprehensive evaluation of patient suitability. By embracing an individualized approach and drawing insights from clinical data, we can move beyond rigid treatment paradigms and tailor transcatheter valve therapy to the unique profile of each patient.

In this way, the so-called “TAVI dilemma” becomes not a problem to solve, but a question to refine—one answered through thoughtful personalization, clinical insight, and the pursuit of better outcomes.

## Figures and Tables

**Table 1:** Comparative overview of Self-Expandable and Balloon-Expandable valves in TAVI procedures across pre-, intra-, and post-procedural phases.

Pre-Procedural Comparison		
Category	Self-Expandable Valve (SEVs)	Balloon-Expandable Valves (BEVs)
Sheath size/Access Compatibility	Smaller delivery systems in patients with small or diseased femoral/iliac arteries (e.g., females)	Slightly larger sheath requirements; may be more challenging in small vessel anatomy
Annular and Anatomical Flexibility	Greater adaptability to irregular or calcified annuli due to flexible frame and consistent radial force	Less forgiving in asymmetric or heavily calcified anatomies; higher reliance on perfect alignment and sizing
Aortic Annulus Size (Small Annuli)	Supra-annular leaflet position allows better flow in small annuli, reducing PPM risk	Intra-annular design may result in higher gradients in small annuli
Native AR	Some SEVs (e.g., JenaValve) specifically designed for pure AR with no calcification for anchoring	Not suitable for non-calcified AR; anchoring is insufficient
Coaxiality/Horizontal Aorta	Navigation may be more difficult in horizontal aortas	Balloon system allows better coaxial alignment in angulated aortic anatomies
Future Coronary Access	High gram height may impede coronary reaccess in ViV or future PCI	Shorter frame height enables easier coronary access post-TAVI (ideal for young or CAD patients)
Bicuspid AV	Less radial strength; potentially higher PVL	Higher radial force improves annular apposition and lowers PVL
Stroke Prevention	Longer manipulation may increase embolic risk (more common in early-generation SEVs)	Shorter deployment reduces risk of embolization
Learning Curve/Operator Experience	Slightly steeper learning curve due to repositioning/retrieval capability	Shorter learning curve, favored for newer centers or less experienced operators
**Intra-Procedural Comparison**		
**Category**	**Self-Expandable Valve (SEVs)**	**Balloon-Expandable Valves (BEVs)**
Repositioning During Deployment	Allows partial deployment, repositioning and recapture if positioning is suboptimal—particularly helpful in complex anatomies	One-shot deployment with NO option of repositioning; accurate initial placement is critical
Need for Rapid Pacing	Rapid pacing not always required, which may reduce hemodynamic stress, particularly in patients with low ejection fraction	Requires rapid ventricular pacing to stabilize the valve and minimize motion during balloon inflation, increasing risk in frail patients
Hemodynamic Stability During Implantation	Maintains better stability throughout the procedure due to self-expanding nature and gradual positioning	Rapid pacing and balloon inflation can lead to transient hypotension or bradycardia
Risk of Annular Rupture	Still present, especially in oversizing scenarios, but lower than BEVs due to less forceful expansion	Higher risk when valve is oversized, especially in heavily calcified annuli
Visualization and Implantation Feedback	Stepwise deployment allows real-time assessment of valve position and function before full release	Limited time for feedback; results are seen only after full deployment
Procedure Time	Slightly longer due to stepwise deployment and need for careful manipulation	Generally shorter due to one-step, balloon-assisted delivery
Coronary Obstruction Mitigation (Real-Time assessment)	Valve can be partially deployed, coronary flow assessed, and recaptured if obstruction is noted before full release	Cannot assess for coronary obstruction before final deployment
**Post-Procedural Comparison**		
**Category**	**Self-Expandable Valve (SEVs)**	**Balloon-Expandable Valves (BEVs)**
Hemodynamic Performance (EOA, Gradient)	Larger EOA, lower mean gradient, and improved flow—especially beneficial in small annuli	Slightly higher residual gradients due to intra-annular leaflet position; often not clinically significant
Durability and Structural Valve Deterioration	Lower gradient rise over time and reduced risk of SVD vs. BEVs over 5 years	RESILIA tissue in newer SAPIEN (Edwards Lifesciences) platforms improves resistance to calcification, enhancing long-term durability in younger patients
Paravalvular Leak	Early-generation SEVs had higher PVL; newer SEVs (e.g., Evolut PRO+—Medtronic Inc., Minneapolis, MN, USA) feature skirts/wraps to reduce moderate/severe PVL	Lower rates of moderate/severe PVL in SAPIEN 3 (Edwards Lifesciences) due to external sealing skirt
Conduction Disturbance/Pacemaker Need	Higher rates of conduction abnormalities and permanent pacemaker implantation due to deeper frame and proximity to conduction system	Three- to four-fold lower risk of pacemaker requirement—less impact of membranous septum and better for patients prone to conduction issues
Bleeding and Vascular Complications	Lower major/life-threatening bleeding due to smaller sheath sizes and reduced access site trauma	Slightly higher bleeding risk due to larger delivery systems, particularly in patients with small vasculature
Mortality (Short- and Mid-term)	Some observational data suggest slightly higher short-term mortality; no significant difference in long-term survival vs. BEVs (CHOICE trial, FRANCE-TAVI)	Lower in-hospital and 2-year mortality shown in large registries like FRANCE-TAVI; possibly related to lower PVL, stroke, and pacemaker rates

Abbreviations: *AR*, Aortic Regurgitation; *AV*, Aortic Valve; *BEVs*, Balloon-Expandable Valves; *CAD*, Coronary Artery Disease; *EOA*, effective orifice area; *PCM*, pacemaker; *PVL*, paravalvular leak; *PPM*, patient-prosthesis mismatch; *PCI*, percutaneous coronary intervention; *SEVs*, Self-Expandable Valve; *SVD*, Structural Valve Deterioration; *TAVI*, transcatheter aortic valve replacement; *ViV*, Valve-in-Valve.

**Table 2:** Details of ongoing studies comparing BEVs and SEVs.

Study	Population	Sample Size	Comparison	Primary Outcome	Main Secondary Outcomes	Completion Date
BASELINE (NCT04843072)	Failing surgical aortic bioprosthesis requiring valve replacement and eligible for transfemoral TAVI	400	Evolut R/PRO (Medtronic Inc., Minneapolis, MN, USA) vs. Sapien S3/Ultra (Edwards Lifesciences)	Device success according to modified VARC-2 (30 d)	All-cause death, disabling stroke, rehospitalization for heart failure or valve related problems (1 y)	1 May 2026
BEST (NCT05454150)	Severe, calcific, symptomatic AS	1862	Evolut R/PRO (Medtronic Inc., Minneapolis, MN, USA) vs. Sapien S3/Ultra (Edwards Lifesciences)	ACM (90 d)	Technical success (post-TAVI) Device success (90 d) Early safety (90 d) ACM (1 y) Stroke/TIA (1 y) HHF (90 d, 1 y)	19 July 2025
CENTER (NCT03588247)	Transfemoral TAVI	12000	NR	ACM (30 d) Stroke (30 d)	PM implantation (30 d) Bleeding (30 d) New-onset AF (30 d) MI (30 d)	1 January 2023
All Women Comparing Self-expanding ALLEGRA Valve to Any Other Balloon-Expandable Valve (NCT05989074)	Female patients with symptomatic severe AS	130	ALLEGRA TAVI System TF vs. any kind of CE-marked BEV system	Trans-aortic MG measure by TTE (30 d)	Technical success (exit the procedure room) Device success (30 d) Early safety (30 d) Clinical efficacy (1 y)	31 August 2025
AAD-CHOICE (NCT06009588)	Severe AS with ascending aorta 45–55mm	100	NR	ACM (30 d) Adverse aortic events (aortic death, aortic dissection, or aortic rupture, 30 d) Device success (30 d)	ACM (1 y) CVM (1 y) Ascending aortic diameter expansion rate ≥ 3 mm/year (1 y) Hospitalization (1 y)	30 August 2026

Abbreviations: *ACM*, all-cause mortality; *AF*, atrial fibrillation; *AR*, aortic regurgitation; *AS*, Aortic Stenosis; *BEV*, Balloon-Expandable valve; *CVM*, cardiovascular mortality; *d*, days; *HHF*, hospitalization for heart failure; *MG*, mean gradient; *MI*, myocardial infarction; *NR*, not reported; *PM*, pacemaker; *PPM*, patient-prosthesis mismatch; *TAVI*, transcatheter aortic valve replacement; *TIA*, transient ischemic attack; *TTE*, Transthoracic echo; *VARC-2*, Valve Academic Research Consortium-2; *y*, year.
